# Supplementary material for: Depressive symptoms and suicidal ideation associated with women’s experience of recent economic intimate partner violence in Gauteng, South Africa: A cross-sectional study
Source: PLOS Glob Public Health. 2025 Dec 22;5(12):e0005390. doi: 10.1371/journal.pgph.0005390 (PMC12721507; doi:10.1371/journal.pgph.0005390)
Supplement: Attachment — Submitted filename: renamed_ca1f9.docx [file pgph.0005390.s002.docx]

## Review of "Depressive symptoms and suicidal ideation associated with women’s experience of recent economic intimate partner violence in Gauteng, South Africa: A cross-sectional study"

## Summary

This manuscript examines the association between economic intimate partner violence (IPV) and women’s mental health (depression and suicidal ideation) in Gauteng, South Africa, using secondary analysis of a population-based survey (n=491). The study highlights an often-overlooked form of IPV and shows strong associations with adverse mental health outcomes, particularly among young women (18–24 years). The research addresses a timely public health concern and contributes important evidence to a limited literature base.

## Strengths

- Focuses on **economic IPV**, an under-researched but critical dimension of IPV.
- Uses **population-based data**, enhancing generalizability compared to prior shelter- or intervention-based studies.
- Applies **validated measurement tools** (CES-D, WHO IPV instrument, CTQ, SRPS).
- Adjusts for **relevant confounders**, including other IPV forms, trauma, and socio-demographics.
- Provides clear **policy and programmatic implications**, especially around youth-friendly GBV and mental health services.

## Major Issues

1. **Age of data**: The dataset is from 2010. While the analysis is novel, the age of the data should be explicitly acknowledged, as IPV dynamics, policy context, and service availability may have changed since then.
2. **Measurement of suicidal ideation**: Suicidal ideation was assessed with a single item tied specifically to violence exposure, which may underestimate broader suicidality. This limitation should be highlighted more clearly.
3. **Causality**: As a cross-sectional study, the analysis cannot establish causality. Phrasing such as “doubles the risk” should be reframed to “associated with higher odds.”
4. **Statistical power**: The relatively low prevalence of economic IPV (9%) may reduce power and precision in some estimates, as reflected in wide CIs. This limitation should be discussed more transparently.
5. **Generalisability**: Gauteng is diverse but may not represent all South African provinces. The authors should temper statements that generalize findings to “South African women” overall

## Minor Issues

- **Abstract**: Include a brief note about key limitations (cross-sectional design, dated data, single-item suicidal ideation measure).
- **Introduction**: The global IPV statistics section could be shortened to sharpen focus on the gap in economic IPV research.
- **Results**: Tables are dense, especially Table 2. Consider simplifying or moving some details to supplementary material. Avoid repeating numbers in both text and tables.
- **Discussion**: At times the tone implies causality (e.g., “economic IPV doubles the risk”). Reframe consistently as “associated with.” Strengthen acknowledgment of data age. Policy implications are valuable but could be presented more concisely.
- **Language**: Some long sentences and minor grammatical errors. A careful language edit is recommended.
- **References**: Ensure formatting consistency per PLOS guidelines.

## Suggested Rewordings for Authors

- Abstract/Results:
  - Current: *“Economic IPV doubled the risk for depressive symptoms and quadrupled the risk for suicidal thoughts.”*
  - Suggested: *“Economic IPV was associated with approximately twofold higher odds of depressive symptoms and nearly fourfold higher odds of suicidal thoughts.”*
- Discussion:
  - Current: *“Economic IPV detrimentally impacts women's mental health.”*
  - Suggested: *“Economic IPV shows strong associations with adverse mental health outcomes.”*
- Policy implications:
  - Current: *“Findings underscore the necessity of sustained economic empowerment and interventions enhancing women’s agency.”*
  - Suggested: *“Findings underscore the importance of sustained economic empowerment and agency-enhancing interventions, while recognizing that the evidence reflects cross-sectional associations.”*
- Limitations:
  - Suggested addition: *“A limitation of this study is the use of survey data collected in 2010. While the analysis provides valuable insights, changes in IPV dynamics, policy responses, and service availability over the past decade may affect the current applicability of prevalence estimates.”*

## Recommendation

**Major Revision** – This study addresses an important and neglected aspect of IPV and provides valuable insights. However, revisions are needed to strengthen framing around limitations, clarify causal language, and refine presentation.

## Confidential Comments to the Editor

This is a relevant and important manuscript with strong potential to advance understanding of economic IPV and its links to women’s mental health. The novelty lies in the use of population-based data, which is a strength compared to most prior studies.

However, I recommend **major revision** due to:

- The age of the dataset (2010), which must be acknowledged more directly.
- The limited measurement of suicidal ideation (single-item, tied to violence).
- The need to avoid causal language in a cross-sectional study.
- Modest prevalence of economic IPV, which limits statistical power.
- Lengthy presentation that would benefit from tightening.

With these revisions, the paper will be a strong contribution to the literature and well-suited for publication in *PLOS Global Public Health*.
