# Supplementary material for: Depressive symptoms and suicidal ideation associated with women’s experience of recent economic intimate partner violence in Gauteng, South Africa: A cross-sectional study
Source: PLOS Glob Public Health. 2025 Dec 22;5(12):e0005390. doi: 10.1371/journal.pgph.0005390 (PMC12721507; doi:10.1371/journal.pgph.0005390)
Supplement: Attachment — Submitted filename: Editorial and Reviewer Comments Plos Global PH 10102025.docx [file pgph.0005390.s004.docx]

| **Editorial and Reviewer Comments** | **Author responses** | **Manuscript Reference** |
| --- | --- | --- |
| Journal Requirements:  1. We noticed you have some minor occurrence of overlapping text with the following previous publication(s), which needs to be addressed:  https://www.emerald.com/books/edited-volume/10989/chapter-abstract/80739948/Examining-the-Promise-and-Delivery-of-Sustainable?redirectedFrom=fulltext  In your revision ensure you cite all your sources (including your own works), and quote or rephrase any duplicated text outside the methods section. Further consideration is dependent on these concerns being addressed. | We have revised the references as per journal guidelines | References page 17 |
| 2. Please amend your detailed Financial Disclosure statement. This is published with the article. It must therefore be completed in full sentences and contain the exact wording you wish to be published.  i. State the initials, alongside each funding source, of each author to receive each grant.  ii. State what role the funders took in the study. If the funders had no role in your study, please state: “The funders had no role in study design, data collection and analysis, decision to publish, or preparation of the manuscript.” | We have added the Financial Disclosure statement | Page 18 Lines 435-438 |
| 3. Please ensure that your Ethics Statement is available in its entirety at the beginning of your Methods section, under a subheading 'Ethics Statement'. | We have revised the subheading to Ethics Statement | Pages 9-10  Lines 213-223 |
| 4. Please upload separate figure files in .tif or .eps format. Also, remove the figures from your manuscript file but keep the legends.  For more information about figure files please see our guidelines:  https://journals.plos.org/globalpublichealth/s/figures  https://journals.plos.org/globalpublichealth/s/figures#loc-file-requirements | We have uploaded Figure 1 separately and kept the legend in the manuscript |  |
| 5. We have noticed that you have uploaded Supporting Information files, but you have not included a list of legends. Please add a full list of legends for your Supporting Information files after the references list. | We added the list of legend on supporting information i.e the minimal dataset |  |
| 6. We note that there is identifying data in the Supporting Information file ‘Depression suicidal economic IPV minimal 20082025.xls’. Due to the inclusion of these potentially identifying data, we have removed this file from your file inventory. Prior to sharing human research participant data, authors should consult with an ethics committee to ensure data are shared in accordance with participant consent and all applicable local laws. Please remove or anonymize all personal information (<specific identifying information in file to be removed>), ensure that the data shared are in accordance with participant consent, and re-upload a fully anonymized data set. Please note that spreadsheet columns with personal information must be removed and not hidden as all hidden columns will appear in the published file.  If the reviewer comments include a recommendation to cite specific previously published works, please review and evaluate these publications to determine whether they are relevant and should be cited. There is no requirement to cite these works unless the editor has indicated otherwise. | We have removed potentially identifying data from the minimal dataset | Supporting information 1 |
| Reviewer #1: This manuscript addresses an important but understudied topic: economic IPV and its association with depressive symptoms and suicidal ideation among women in Gauteng, South Africa. Strengths include use of population-based data, validated tools, and policy-relevant implications. However, I recommend major revision. Key concerns: (1) the dataset is from 2010 and this limitation must be emphasized; | Thanks for the helpful feedback. To address the concerns raised we have:   - Added the date of data collection in Abstract section - We added the date of data collection in Methods section - We added text to highlight the study limitations dues to the date of data collection which is 2010 and justified the usefulness of the findings for answering the critical research questions | Page 2 Line 20  Page 6 Line 136  Page 15-16 Lines 386-395 |
| (2) suicidal ideation was measured with a single item tied to violence, which likely underestimates prevalence; | The measurement of suicidal ideation was not tied to experiences of violence. However, we added the following study limitations:  *Suicidal ideation was assessed using a single item may have led to an underestimation of its true prevalence* | Page 16 Lines 402-403 |
| (3) causal phrasing (“doubles the risk”) should be revised to reflect associations; | Thank you for your valuable feedback. We have carefully revised the results and discussion sections to remove any language implying causality. All statements now accurately describe the findings as associations between economic IPV and mental health outcomes. | All through manuscript |
| (4) low prevalence of economic IPV limits statistical power and precision; | We have noted this limitation as follows:  *Moreover, the relatively low prevalence of past-year IPV within the sample may have limited the study's statistical power to detect associations and estimate effect sizes with greater precision.* | Page 16 Lines 400-402 |
| and (5) conclusions should not overgeneralize beyond Gauteng. | Thank you for your thoughtful feedback. We have been careful to qualify the study implications as applying specifically to Gauteng Province, South Africa. At the same time, we have contextualized the findings within the broader South African setting, noting that Gauteng is the most populous of the nine provinces, which suggests that the observed associations may be relevant to a substantial proportion of the country’s female population. We hope these changes address your concerns satisfactorily. | Abstract Page 2 Line 34 and Conclusion Page 16 Line 412 |
| The introduction and discussion could be streamlined, tables simplified, and language edited. With these revisions, the study would make a valuable contribution. | Thank you for your thoughtful feedback. We hope the changes we made address your concerns satisfactorily. |  |
| Reviewer #2: The study addressed an important gap in understanding economic intimate partner violence and its mental health impacts using population-based data from South Africa. The strengths of the paper include: The secondary analysis is appropriate for exploring the research questions using existing population-based data, multi-stage randomized sampling design provided good representativeness for Gauteng Province, the use of validated instrument (CES-D for depression, WHO questionnaire for IPV) is methodologically sound, appropriate statistical approach with multivariate logistic regression controlling for relevant confounders, and good response rate (75% overall, 73% for women). | Thanks for the positive feedback on the article |  |
| The abstract does not adhere to the journal’s guidelines. It exceeded the 300-word limit and uses subheadings. Please revise it to be a single, concise paragraph of not more than 300 words, containing the required information (background, objectives, methods, results and conclusion). | We have revised the abstract to adhere to journal guidelines | Abstract Page 2 |
| 2. In the abstract section, the data collection date (the survey year, 2010) should be mentioned upfront for transparency. | We added the data collection date to the abstract and methods section | Page 2 Line 20  Page 6 Line 136 |
| 3. Data from 2010 is now 15 years old – IPV patterns and mental health service availability may have changed significantly. The authors may wish to discuss limitations and current relevance | We discussed the datedness of the data as follows:  *The findings of this study should be interpreted in light of certain limitations. One notable limitation is the use of survey data collected in 2010. Since this period, only a limited number of population-based surveys measuring gender-based violence (GBV) in South Africa have been conducted, with the recent completion of the first national GBV survey being a major advancement (5). The 2010 data, drawn from the most populous of South Africa’s nine provinces, provided a valuable opportunity to interrogate the research questions and generate important insights at that time. However, the ongoing analysis of the recently completed national survey will be critical to understanding how changes over the past decade—in women’s economic empowerment, GBV policy frameworks, prevention programming, and service availability—have influenced the prevalence of women’s experiences of economic intimate partner violence (IPV) and their associations with mental health observed in this study.* | Page 15-16 Lines 386-395 |
| 4. Age categorisation seems arbitrary (18-24, 25-34, 35-49, 50+) without justification (Table 2). The authors should either provide a rationale for age categorisation or use standard epidemiological age groups. | Thank you for your comment. Age was collected in predefined categories as part of the primary survey design, and we were unable to change or recode these categories in the secondary data analysis. We therefore adopted the same categorical age groups used in the original study |  |
| 5. The manuscript used causal language (e.g., increased risk – line 307 and 356, heightened risk – line 384) in the results and discussion sections. However, in a cross-sectional study, it can only demonstrate associations, not causality. The authors may wish to revise the language throughout the manuscript to reflect association rather than causation. Alternative phrasing:  a. Change “A increases the risk of B” to “A is associated with B.”  b. Change “A leads to B” to “A is linked to B.” | Thank you for your valuable feedback. We have carefully revised the results and discussion sections to remove any language implying causality, ensuring all statements accurately describe the findings as associations between economic IPV and mental health outcomes. However, in the background section, we have retained the use of terms such as "risk factors" and "increasing risk" as applied in the cited literature, which appropriately reflect terminology used in temporal studies and observational longitudinal studies. |  |
| 6. Table 2 (Line 567) shows 24.2% with probable depression, but the abstract says “twenty-four per cent” – these should match exactly. Some percentages in tables don’t align with the stated sample size | We have edited the abstract so that the prevalence reported in the abstract matches in Table 2. | Abstract Page 2 |
